# Similarity-based gene detection: using COGs to find evolutionarily-conserved ORFs

**DOI:** 10.1186/1471-2105-7-31

**Published:** 2006-01-19

**Authors:** Bradford C Powell, Clyde A Hutchison

**Affiliations:** 1 Curriculum in Genetics and Molecular Biology, University of North Carolina at Chapel Hill, Chapel Hill, North Carolina, USA; 2 Department of Microbiology and Immunology, University of North Carolina at Chapel Hill, Chapel Hill, North Carolina, USA; 3 J. Craig Venter Institute, Rockville, Maryland, USA

## Abstract

**Background:**

Experimental verification of gene products has not kept pace with the rapid growth of microbial sequence information. However, existing annotations of gene locations contain sufficient information to screen for probable errors. Furthermore, comparisons among genomes become more informative as more genomes are examined. We studied all open reading frames (ORFs) of at least 30 codons from the genomes of 27 sequenced bacterial strains. We grouped the potential peptide sequences encoded from the ORFs by forming Clusters of Orthologous Groups (COGs). We used this grouping in order to find homologous relationships that would not be distinguishable from noise when using simple BLAST searches. Although COG analysis was initially developed to group annotated genes, we applied it to the task of grouping anonymous DNA sequences that may encode proteins.

**Results:**

"Mixed COGs" of ORFs (clusters in which some sequences correspond to annotated genes and some do not) are attractive targets when seeking errors of gene predicion. Examination of mixed COGs reveals some situations in which genes appear to have been missed in current annotations and a smaller number of regions that appear to have been annotated as gene loci erroneously. This technique can also be used to detect potential pseudogenes or sequencing errors. Our method uses an adjustable parameter for degree of conservation among the studied genomes (stringency). We detail results for one level of stringency at which we found 83 potential genes which had not previously been identified, 60 potential pseudogenes, and 7 sequences with existing gene annotations that are probably incorrect.

**Conclusion:**

Systematic study of sequence conservation offers a way to improve existing annotations by identifying potentially homologous regions where the annotation of the presence or absence of a gene is inconsistent among genomes.

## Background

The rapidly growing amount of genomic sequence information necessitates tools for its annotation. Although predicting bacterial genes is in many ways simpler than predicting eukaryotic genes, it is clear that there remains room for improvement in the bacterial case. Several groups have undertaken efforts to re-annotate specific genomes [1-3], often finding a small but significant number of errors in existing annotation of gene loci. The presence of these errors has motivated the effort of some groups to systematically revise the gene annotations in public databases as a continuous process [4,5].

Because technology for genome sequencing is much more mature than proteomic analysis, only a small fraction of annotated bacterial gene products have been detected as protein; most have been annotated using only computational methods. Although methods for detecting and identifying all proteins in a cell are being developed [3,6,7] and incorporated into annotations of newly-sequenced genomes [8], these techniques are currently limited by the ability to express all of the polypeptides in an organism and separate them into fractions with low enough complexity for analysis. It is still useful to refine our computational predictions so that we can make targeted searches for potential proteins.

Accuracy of gene identification is particularly important in studies of the gene content of a genome as a whole. Studies of phyletic patterns of gene presence [9], the extent of horizontal gene transfer among genomes, the entire set of protein structures encoded by a genome [10], and the components of a "minimal genome" [11,12] are all predicated on an accurate catalog of the genes within an organism. Because these studies involve comparing the presence or absence of genes among several organisms, it is particularly important that all of the genes present be identified. Insights in these areas of study could impact our understanding of bacterial evolution physiology and pathogenicity. As an example, in the initial report of the *Mycoplasma mobile *genome sequence the correlation of presence or absence of certain genes with a presence or absence of a specific phenotypic characteristic (motility) among nine species was used to suggest genes which might confer that phenotype [8].

Methods for predicting protein-coding genes are often divided into *intrinsic *and *extrinsic *classes [13,14]. Intrinsic methods only use evidence from within the primary sequence of a genome. This evidence may include i) the presence of a relatively long frame uninterrupted by a stop codon, ii) the statistical pattern of polynucleotide stretches that match the typical frequencies present in other coding regions of the organism and iii) the existence of appropriate non-coding control elements. It may be difficult to identify some small genes using the first two types of evidence; small genes can be difficult to distinguish from open reading frames that occur by chance, and in such short regions, sequence characteristics may be affected stochastically. Gene finding methods which use sequence characteristics or control elements often need to be tuned for the specific organism studied, and in many cases several statistical models of coding regions may need to be developed in a single organism [15].

Extrinsic methods use information from comparisons of genomes. These analyses originally used simple pairwise comparisons among potential protein-coding regions. Harrison et al. [16] examined ORFs of 15 or more codons in 65 microbial genome sequences, using BLAST E-value of less than 10^-4 ^to indicate similarity suggestive of conserved function. Other searches use sequence alignments of a protein family as a query against all possible translations of the genome of interest. This alignment may be specified beforehand (e.g. using a Pfam protein family) or developed as part of the search as by PSI-BLAST [17]. Pair hidden Markov models use a pairwise sequence alignment coupled to a hidden Markov model to more precisely determine the amino termini of protein-coding genes [18]. Programs have been developed that use pairwise alignment of syntenic regions to predict gene structure in eukaryotes [19]. The ratio of synonymous to nonsynonymous substitutions between pairs of putative genes can be used to examine whether there is selection for protein-coding function, but this requires sequences from closely related organisms [20]. The majority of bacterial genome annotations have used intrinsic methods at least initially to predict the presence of genes. Extrinsic gene prediction methods serve as a useful complement to intrinsic methods because independent information is used to make the same prediction.

In this study we describe a systematic extension to examining similarity shared among several genomes using a modification of the analysis of Clusters of Orthologous Groups (COGs). COGs were developed to cluster annotated genes into functionally related groups in order to facilitate the transfer of functional annotations among organisms [21]. Here we use COGs to cluster open reading frames as a means of recognizing genes. An advantage of the COG analysis is that no explicit threshold for sequence similarity is used; genes that are missed in pairwise comparisons may be detected. Because the focus is on annotation of gene location rather than gene function, we are not concerned with finding genes that are strictly orthologous. Evidence of homology to another gene is sufficient to imply that a region is a gene, and we make no effort to avoid the joining of COGs which may occur due to a gene fusion [22]. The idea of stringency [23] of COGs expands upon the initial COG definition by requiring an adjustable of connectedness for grouping genes. As the number of studied organisms increases, the stringency can further filter some similarities which may have occurred by chance. ORFs that do not correspond to annotated genes but that nonetheless have conserved sequences present in several genomes are likely to be protein-coding genes that have been missed by current annotations.

A cluster of ORFs can be examined with regards to its multiple sequence alignment, the network of similarities among the ORFs, and the respective genomic contexts of the ORFs in the cluster. These characteristics of ORF clusters can be used to screen existing gene predictions for potential errors. The extrinsic nature of the use of COGs of ORFs is complementary to the intrinsic methods that have been used in producing the majority of gene loci annotations. Because our strategy has different strengths and weaknesses from the intrinsic methods, it may be expected to pick up some genes that other methods have missed.

In this study we examined open reading frames from the complete genome sequences of 27 bacteria (Table 1). We selected these genome sequences for the following reasons. *M. genitalium *and *M. pneumoniae *are model systems for defining the minimal cellular genome. Consequently, their proteins became the focus of research at the Berkeley Structural Genomics Center [10,24]. Other members of the class Mollicutes provide a closely related set which may help identify genes specific to this class. The protein products of *M. pneumoniae *and *M. mobile *have been recently studied using high-throughput identification using multidimensional chromatography and mass spectrometry [3,8]. We included other small-genome parasitic bacteria to study the extent to which gene content has convergently evolved among this group. We also included a more diverse selection of bacterial genome sequences from major phylogenetic groups to see how robust our strategy would be when examining distantly related organisms.

We wanted to detect fragments of genes and genes which use start codons other than ATG, so we used a very general definition of an ORF: any frame of length at least 30 codons (90nucleotides) uninterrupted by stop codons. The software developed as part of this study, SPROCKET, can be used to detect probable errors in existing gene annotations.

## Results

### Gene discovery using conservation of potential peptide sequence

Extrinsic gene-finding methods are based on the duality that, given sufficient evolutionary distance, conserved sequences are likely to be functional and that functional sequences are likely to be conserved. The conserved regions may represent control elements or may encode functional RNA molecules or proteins. We examined the extent to which multi-species sequence conservation could be used to detect the presence of protein-coding genes. We considered two systems for classifying ORFs as genes or not-genes. The first system was based solely on existence of a COG containing the ORF. If we determined that an ORF was a member of a COG then we classified it as a gene, otherwise we classified it as not being a gene. Our second classification system used existing gene annotations for all genomes except the one containing the ORF to be classified, reducing the number of false positives. In this system we classified an ORF as a gene if we found it in a COG containing at least one ORF from another genome that was annotated as a gene. In both systems the COG stringency controlled the extent of conservation required for classification.

We compared these gene predictions to existing gene annotations using sensitivity/specificity analysis. Although there are errors in existing gene annotations (as discussed below) the current annotations represent the expert consensus. Sensitivity for a gene classification system is the number of correct gene predictions divided by the number of actual genes. The specificity is the number of true negative predictions divided by the total number of non-genes.

**Figure 1:**
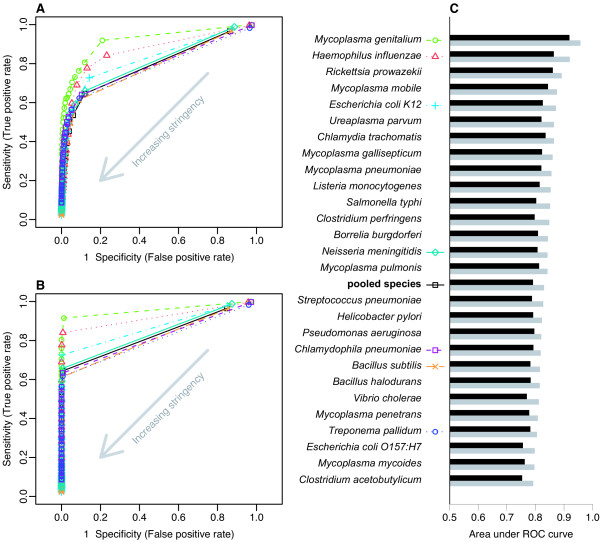
**Gene prediction based on sequence conservation**. (A) and (B) show receiver-operator characteristic curves summarizing the sensitivity and specificity of gene prediction based on COG membership when compared to the current gene annotations. In (A), an ORF is classified as a gene if it is conserved in a COG at a certain stringency; for (B), the ORF must be in a COG that contains at least one annotated gene from another species. Curves are produced by examining COGs at different stringencies. At stringency 2, tests are very sensitive but not very specific (points at upper right of each panel). As stringency increases, specificity increases and sensitivity decreases (indicated by arrow). For clarity, full ROC curves are shown for only seven of the organisms studied, and for the pooled result among all of the organisms studied. The plotting symbols and colors used in (A) and (B) are next to the organism names in (C). (C) shows the areas under the curves in (A) black bars and (B) grey bars. The ROC curve of a perfect test would enclose an area of 1, for a completely arbitrary test the area would be 0.5. The organisms in (C) are ordered by the area under the ROC curve in (B).

Sensitivity and specificity of gene predictions varied at different stringency levels of the COGs used in classification. The accuracies of both classification systems are summarized in Figure 1. Receiver-operator characteristic (ROC) plots show the true positive rate (sensitivity) along the vertical axis against the false positive rate (1-specificity) along the horizontal axis. An ideal test (when compared to an ideal "gold standard") would be represented by a point plotted in the upper-left corner (0,1). Figures 1A and 1B show the classifications based solely on COG membership and based on COG membership with an annotated gene from another organism, respectively. Each point plotted on this graph represents the sensitivity and specificity of classification at a given stringency. ROC curves are shown for a sample of seven of the genome sequences studied and for the pooled results for all of the genomes. The accuracy of tests for each genome sequence and pooled results for all of the genome sequences are summarized across COG stringencies by the area under the ROC plots (Figure 1C). The sensitivity and specificity values for all of the studied genomes were computed [see Additional file 1].

### "Mixed" COGs

**Figure 2:**
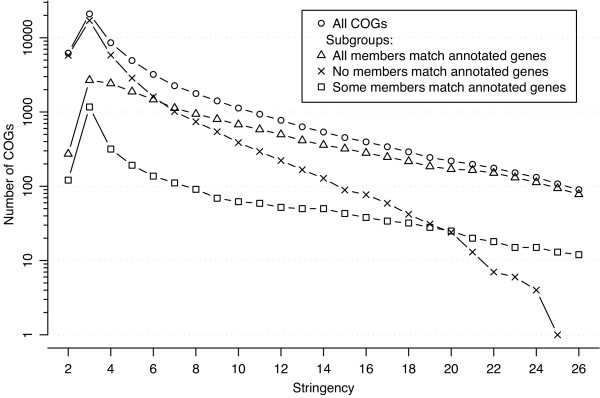
**Presence of annotated genes in COGs of ORFs**. Open reading frames (ORFs) of at least 90 nucleotides between stop codons were used to construct COGs at varying stringencies as described in the methods. COGs were divided into one of three groups: "All members match annotated genes" – contain only ORFs which correspond to annotated genes, "No members match annotated genes" – contain no ORFs which match annotated genes, or "Mixed" – contain some ORFs that correspond to annotated genes and some that do not. The numbers of ORFs in COGs of each of these classes are plotted along the y-axis with a logarithmic scale.

COGs formed from ORFs can also be used to mine existing gene location annotation for potential errors. COGs in which there are some ORFs that correspond to annotated genes and other ORFs that do not correspond to annotated genes represent potential anomalies in existing annotations. The number of these mixed COGs at different stringencies is shown in Figure 2. Also shown in Figure 2 are the number of COGs at each stringency level which contain only ORFs which correspond to annotated genes ("all matches") or which contain no ORFs which correspond to annotated genes ("no matches"). The initial increase in numbers of COGs when moving from stringency two (single-linkage clusters) to three is the result of larger, weakly connected COGs splitting into several smaller COGs. As stringency increases beyond three, the number of COGs in each group decreases exponentially. The "all matches" and "mixed" classes have similar connectedness structures in that the numbers of COGs in these classes decay at similar rates. The number of COGs in the "no matches" class drops more rapidly because the less well-conserved or connected sets of ORFs that may not be conserved due to protein-coding function are not present at higher stringencies.

### Screening existing gene predictions for errors

Mixed COGs are attractive targets when looking for errors in existing gene predictions. We examined the genomic context and peptide sequence alignments of the mixed COGs of stringency six to explain the inconsistency of gene annotation within these COGs. In COGs where the majority of ORFs correspond to annotated genes, the remaining ORFs are likely to represent missed genes or pseudogenes. At stringency six there are 147 mixed COGs in which the majority of ORFs correspond to annotated genes (Table 2). At this stringency every member of a COG is in a bidirectional best-hit relationship with at least six other ORFs in the COG. These COGs contain 143 ORFs that are not associated with annotated genes. Some of the COGs contained multiple ORFs that did not correspond to annotated genes and some ORFs were members of multiple COGs.

The potential amino acid sequences of 83 of these ORFs contain regions that have substantial similarity to the multiple-sequence alignment of the annotated genes that are also members of the COG (Table 2). We judge that these ORFs are likely to represent genes missed in current annotations. 5 of the 83 candidate genes involve instances where there were two ORFs with approximately equal length present in two different strands; however the opposite strand had previously been chosen as coding.

In 60 (of 143) cases comparison of gene lengths to ORF lengths indicates that the ORFs that are not annotated as genes may be pseudogenes (Table 3); most have frameshifts but some have nonsense mutations. 20 of these are annotated as pseudogenes in Genome Reviews 25.0 [4,25]. It is possible that some of the apparent nonsense or frameshift mutations may be due to sequencing errors.

For COGs in which the majority of members do not correspond to annotated genes, the preponderance of evidence suggests that sequence conservation can be better explained by reasons other than protein coding of the ORF. At stringency 6 there are 12 of this type of COG among the organisms we studied, representing 11 distinct annotated genes (Table 4). The presence of a conserved ORF of length sufficient to be a potential gene may be explained by the presence of a gene in the opposite strand. This is particularly likely in species such as those of the *Mollicutes *class that do not use UGA as a stop codon. In these organisms, the exclusion of TAA and TAG in the actual coding frame can be associated with a relative shortage of TAn codons in the opposite strand, leading to ORFs of substantial length on the non-coding strand. This was the case for 7 of the 11, and we predict that the previous annotations are erroneous. Three of the annotated genes in majority-unannotated COGs overlap regions that appear to encode non-translated RNA genes. The constraints of the RNA genes may have reduced the probability of occurrence of stop codons in the region, which lead to ORFs of sufficient length that they have been annotated as hypothetical genes in previous annotations.

Possible pseudogenes as listed in Table 3 can be re-sequenced to evaluate whether there is in fact an underlying sequencing error. Although this could be done on individual regions of a genome, the recent re-sequencing [26] of *Mycoplasma genitalium *strain G-37 [GenBank:AAGX00000000] when compared to the original sequence [NC_000908.1] is illustrative. There are no mixed COGs at stringency 6 which contain unnannotated ORFs from the original *M. genitalium *sequence, but there are four such COGs at stringency 4 (Table 5). One of these ORFs (in COG 4-3347) is highly similar to sequences from 12 other organisms. The other ORFs contain apparent frameshifts that are resolved in the new sequence.

## Discussion

### Peptide sequence similarity as a gene discovery technique

Although studies of intrinsic gene prediction report higher accuracies (for example, GeneMark is reported to have detected genes with sensitivity 98.3% and specificity 91.3% averaged over eight prokaryotes [27], this fact should be considered with the caveat that those algorithms or algorithms similar to them were used to produce the initial annotations. Because intrinsic methods require statistical models of coding sequence to be tuned to a specific organism, they may miss recently acquired genes for which selection has not yet altered the polynucleotide frequencies to match the new host organism. Intrinsic methods may also miss small genes for which there is insufficient nucleotide sequence to provide a statistically significant result. Most of the genes missed by our method are likely to be species specific among the organisms studied, (i.e. they are ORFans [28] within the context of the analyzed genomes). Our method may be more able to pick up genes that may have been horizontally transferred when compared to intrinsic methods.

A different choice of size cutoff for consideration of ORFs would affect the accuracy of our method because longer ORFs are more likely to represent genes but genes smaller than the cutoff would be excluded. An increase in ORF size cutoff would result in an increase in specificity with a corresponding decrease in sensitivity. The fact that we were able to detect some genes that had been missed in prior genome annotations is in part because we used a very small size cutoff for consideration of which ORFs may be genes. The newly detected genes we report are disproportionately small (27% are shorter than 100 codons, compared to 11% of annotated genes).

A comparison of Figures 1A and 1B shows that incorporating gene information from other organisms greatly increases the specificity (reducing false positives) of similarity-based gene identification, with a much smaller decrease in sensitivity. Classification using stringency 2 COGs (i.e. single linkage clusters) is highly sensitive but not very specific. Increasing stringency to three (the COGs as described by Tatusov et al. [21]) causes the largest difference in specificity. The difference in specificity between stringencies two and three is even more pronounced when incorporating gene information from the other organisms.

The two gene prediction systems shown in Figure 1 are in general most accurate for the small genomes (*Mycoplasma genitalium *, *Haemophilus influenzae *, other *Mycoplasma *species). The high accuracies for *Mollicutes *may be explained by the presence of many members of this class in the set of organisms studied. The genes for *Escherichia coli *strain K12 can also be relatively well predicted, perhaps due to the fact that it is so well studied. Classification of *E. coli *genes performs particularly well in the second classification system, when information about annotated genes from other genomes is included. This may be due to the fact that the characteristics of genes of *E. coli *have been used to find those other genes in other organisms.

### Limitations of using sequence conservation for gene prediction

Genes with little conservation among the studied genomes or which are only present in a few genomes cannot be detected using sequence conservation, leading to false negatives. Sequence similarity can be due to reasons other than selection due to protein-coding function. This can lead to false positive gene predictions. Sequences that do not encode protein may be similar because: i) they are conserved for other reasons (non-coding control elements, RNA-genes) or ii) because there is insufficient evolutionary distance between a pair of studied species. The latter can be the case for pseudogenes – a region in one genome may contain a gene while the corresponding region in another genome may not actually code for protein but may not have accumulated enough mutations to prevent detection of homology.

### Examples of gene prediction inconsistencies in COGs of ORFs

The ORFs in *Mycoplasma penetrans *from 1316960 to 1317088 on the minus strand and in *Mycoplasma mycoides *from 830742 to 830915 on the minus strand do not contain annotated genes. They are however members of a COG at stringency 6 (COG id 199 in Table 2) in which the majority of ORFs (25 of 27) correspond to annotated genes. The annotated members of the COG encode the 50S ribosomal protein L36. The sequence identities from the *M. penetrans *ORF to the annotated genes in the COG range from 39.0% to 83.3%. For the *M. mycoides *ORF the range is from 35.2% to 75.7%. Neither of the ORFs have any interruption in coding potential compared to the annotated genes in the COG. It is likely that these ORFs contain genes that were missed in the initial annotations of their organisms. The peptides they encode are less than 40 amino acids long, and this may account for the fact that the genes had not been previously detected.

The *Vibrio cholerae *ORF from 637551 to 638246 in the plus strand of chromosome I is present in a COG of stringency 6 (COG id 1826 in Table 3) in which 11 ORFs correspond to annotated genes. The *V. cholerae *ORF is only about 60% as long as the annotated genes, and the potential peptide sequence it encodes aligns to the amino terminal region of the annotated genes. Examination of the genomic context of the members of this COG reveals a nearby *V. cholerae *ORF from positions 638126 to 638788 which could encode peptide which would align the to carboxy terminus of the annotated genes of this COG. This suggests the presence of a frameshift or sequencing error in the region encompassing the two *V. cholerae *ORFs.

The *secG *gene annotated in *M. genitalium *, *M. penetrans *, *M. pneumoniae *and *U. parvum *has homologs in *M. gallisepticum *, *M. mobile *, *M. mycoides *and *M. pulmonis *. The *M. genitalium *homolog was detected by curators of the Genome Reviews database (it is not identified in the EMBL genome file). This group of homologs is present in COG id 3175 in Table 2. The *M. gallisepticum *homolog is identified as a potential gene, but given the annotation "unique hypothetical". The *M. mobile *and *M. mycoides *homologs are not identified as genes. In *M. pulmonis *, the ORF homologous to *secG *(genomic coordinates 412829 to 413074 on the forward strand) is not identified as a gene, but overlaps the locus *MYPU_3500 *which is on the opposite strand. It is likely that this is an error in the existing annotation – that the *secG *homolog is a real gene and the *MYPU_3500 *locus, while being slightly longer, does not actually encode a peptide sequence in the cell.

### Detecting pseudogenes

Several groups have used computational techniques to find pseudogenes in prokaryotes. Intrinsic techniques are poorly suited for identifying pseudogenes. Pseudogenes may lack characteristics of protein-coding sequence in a particular organism because they have resulted from recent horizontal transfer [29] or because there is a lack of selective pressure to maintain the characteristics. As a result, extrinsic techniques are common when searching for pseudogenes. Liu et al. [29] sought pseudogenes using SwissProt entries as queries in FastX searches against genome sequences. They used a fixed similarity cutoff score of 0.01 to define significant matches. The lack of selective pressure and resulting genetic drift that makes it difficult to find pseudogenes by intrinsic methods can also make it difficult to find homology between a pseudogene and the gene from which it was derived in disparate genome sequences. Lerat and Ochman [30] considered sets of closely related organisms and used thresholds for inferred homology as strict as TBLASTN E-value < 10^-15 ^and protein identity >79%. By using COGs we were able to detect much more distant homology. There were 232 COGs of stringency-6 that contained at least one best-hit with a BLASTP E-value > 1.0.

### Potential extensions and modifications to the methods of this study

One obvious extension of this study is to apply the strategy described here to more genome sequences. The scalability of the methods described in this study are limited primarily by the initial BLASTP searches of translations of the ORFs. Because all pairwise comparisons are performed, this step scales as the square of the number of ORFs among the genome sequences studied, which can be approximated by the square of the number of genome sequences. As of June 2005, there are 211 bacterial and 21 archaeal genome sequences published [31], about 8.9 times the number of genome sequences analyzed here. The similarity search comparison step is time consuming but highly parallel. Because each similarity search is independent of the other searches, the process can be carried out among many CPUs. New genome sequences can be added to the study incrementally.

One shortcut that could mitigate scaling issues would be to form a standard set of COGs of the various stringencies among only the annotated genes. All of the ORFs in a test genome sequence could then be compared to the members of the standard COGs. The annotated genes of the genomes studied here are only 5.75% of the ORFs of 30 or more codons, greatly reducing the number of comparisons that would need to be performed. This shortcut has a few drawbacks: i) it will not be possible to detect genes which are not present in enough of the genomes in the standard set (depending on stringency) and ii) it will not be possible to find the 'minority-annotated' mixed COGs that can indicate over-prediction of genes.

The varying stringency COGs produced using the techniques of this study could also be used in other analyses. The multiple sequence alignments of COGs could be used to help define which of several potential translational start sites may be used in a given gene. Stringency-three COGs which contain members in many organisms have been used to indicate genes which may be essential on account of their pervasiveness. Higher stringency COGs show not only that corresponding elements are present in many organisms but also that between most or all of pairs of organisms the elements are best-hit pairs of each other.

### Relationship to proteomic studies

Jaffe et al. [3] revisited the annotation of genes in *Mycoplasma pneumoniae *, incorporating evidence of peptides detected using multi-dimensional chromatography followed by analysis by mass-spectrometry. They detected evidence for 16 proteins which could not be associated with annotated genes. One of these (from 207448 to 207717 in the (+) strand) was present in COGs up to stringency 10. Two more (from 250021 to 250293 and from 415490 to 416032, both on the (+) strand) were present in COGs of stringency 3. The others were only present in COGs of stringency 2. The Jaffe et al. study was aided by the fact that *M. pneumoniae *has a simple lifestyle and grows in a relatively static natural environment. Although some change in *M. pneumoniae *gene expression is reported in response to heat shock [32], it is thought that most of its genes are expressed constitutively. In organisms with more complex niches or lifestyles that may involve growing in multiple hosts or environments, the shotgun proteomic approach will require exposing the organisms to multiple conditions in order to induce detectable expression of all proteins.

## Conclusion

In this paper, we describe a method that can be used in combination with existing techniques for detecting protein-coding gene sequences in bacterial genomes. Our method is extrinsic an individual in that it incorporates pairwise sequence similarities among several genomes.

The methods we describe can also be used to screen existing gene predictions. The "mixed COGs", in which some open reading frames correspond to annotated genes and some do not, are attractive targets for further study. Such COGs exist even when requiring best-hit similarity pairings among many organisms, and we list the COGs of this type that exist at stringency six among 27 sequenced bacterial strains. The methods we describe can be used to generate hypotheses about the presence of specific genes that may have been missed in existing annotations. Such a hypothesis could be evaluated by a targeted search for the expected protein product based on predicted protein characteristics.

## Methods

### Sequence preparation, comparison and Best Hit determination

Sequences and existing annotations for genomes under study (Table 1) were obtained from the Genome Reviews database [4] version 25.0 [25]. The annotations in the Genome Review database include those from the EMBL sequence files with corresponding accessions as well as some genes that were identified on the basis of BLAST similarity to sequences in UniProt. We located open reading frames (ORFs) using the criterion of at least 90 nucleotides (30 codons) between in-frame stop codons (for codon usage tables appropriate to each organism – the included *Mollicute *species do not use UGA as a stop codon).

Sequence libraries composed of all of the ORFs in the sequences of included genomes were searched using each ORF as a query. These all-against-all searches were performed using WU-BLAST (BLASTP 2.0 MP-WashU [06-Apr-2005] [macosx-10.2-g4-ILP32F64 2005-04-06T17:46:37], BLOSUM62 similarity matrix, filtered with SEG, Smith-Waterman alignment used in the scoring phase). The top ten hits from each query to a library of ORFs from another organism were recorded provided the BLAST e-score was less than or equal to 20. There were 84009520 BLAST hits which met these criteria.

### COG analysis

A modification of the concept of Clusters of Orthologous Groups (COGs) [21,33,34] which includes levels of stringency [23] was used to group similar sequences among organisms. The COGs formed are graphs with ORFs as vertices using the following procedure:

1. For each ORF, compare it to all ORFs in another organism, recording the best hit (BeT), provided that hit meets the loose stringency cutoff mentioned above (e-score less than 20).

2. If an ORF *a *has ORF *b *as its best hit in another organism and *b *has *a *as its best hit when the reciprocal similarity search is performed then *a *and *b *are said to have a "bidirectional" or "congruent" best-hit relationship. A pair of vertices will have edges connecting them if the ORFs they represent have a bidirectional best-hit relationship. For purposes of this step, ORFs the two strains of *E. coli *were not compared to each other.

3. For a given stringency *n *, cliques (complete graphs – wherein all nodes are connected to all other nodes) of size *n *are found. Cliques are joined when they share a sub-clique of size *n-1 *maximally-joined sets of cliques form a COG.

**Figure 3:**
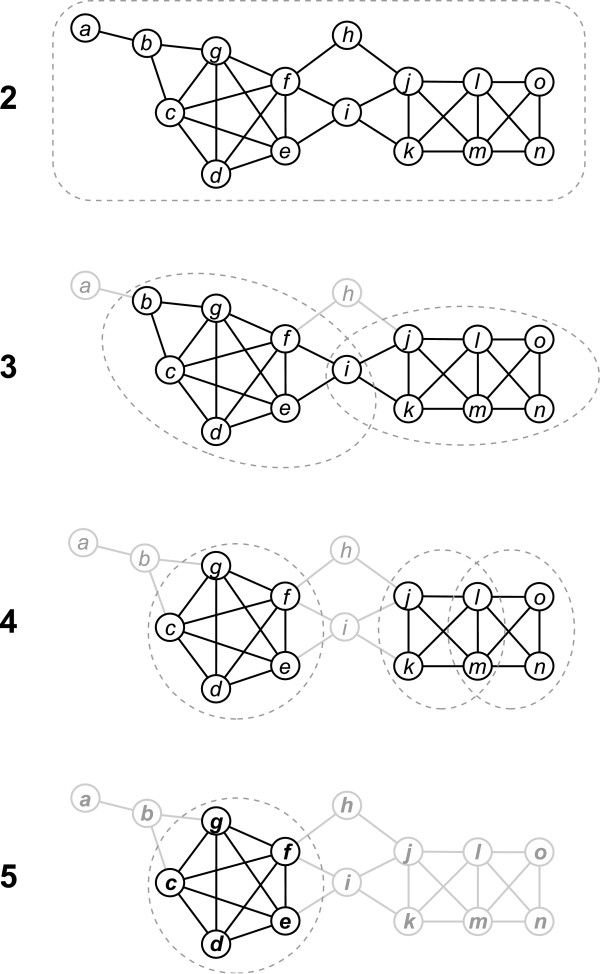
**COGs at varying stringencies**. The concept of stringency places a requirement of interconnectedness of elements of a COG. As stringency increases, COGs may split into smaller COGs and less-connected nodes are dropped. Each vertex represents a gene (as used in the initial definition of COGs) or an ORF (as used in this study). Edges represent bidirectional best-hit pairs. Dashed lines enclose elements of a single COG. Grayed vertices and edges do not participate in a COG at the given stringency. There is a single COG of stringency (2) containing all of the vertices in this graph because they are all transitively connected. Stringency (3) COGs are as described by Tatusov et al. [21]. An orthologous group of stringency 3 forms a triangle (such as {*i *, *j *, *k *}); orthologous groups of stringency (3) are clustered if they share two vertices (alternatively: if they share an edge). Stringency (4) OGs are clustered if they share three vertices. The orthologous groups {*j *, *k *, *l *, *m *} and {*l *, *m *, *n *, *o *} only share two vertices so they form two separate COGs. At stringency (5) only one orthologous group, and thus only one COG, remains.

According to this formalization, the original COGs as described by Tatusov, et al. [21] are COGs of stringency three. They consist of triangles formed from congruent best-hit relationships which are clustered by shared edges. These triangles are cliques of size 3 and are referred to by Tatusov et al. as orthologous groups (OGs). Stringency-two COGs are equivalent to graphs clustered by single linkage. Each stringency-two COG is an individual connected component of the total graph. As stringency increases, poorly connected vertices drop out of COGs and COGs may split (Figure 3).

Like the extended COGs used in the STRING database [35], the COGs in this study are 'non-supervised'; we have not performed any manual curation subsequent to COG production. A gene fusion may result in the merging of two disparate COGs. Although such an artifact affects the use of COGs in functional annotation, it is not problematic when using COGs for gene recognition so we made no effort to avoid these merging events. The BLAST searches resulted in 2649524 best-hit pairs involving 891039 ORFs. Only 38 best-hit pairs involved a similarity with E-value as high as 20, and none of these best-hit pairs held together a COG of stringency three or higher.

#### SPROCKET

**Figure 4:**
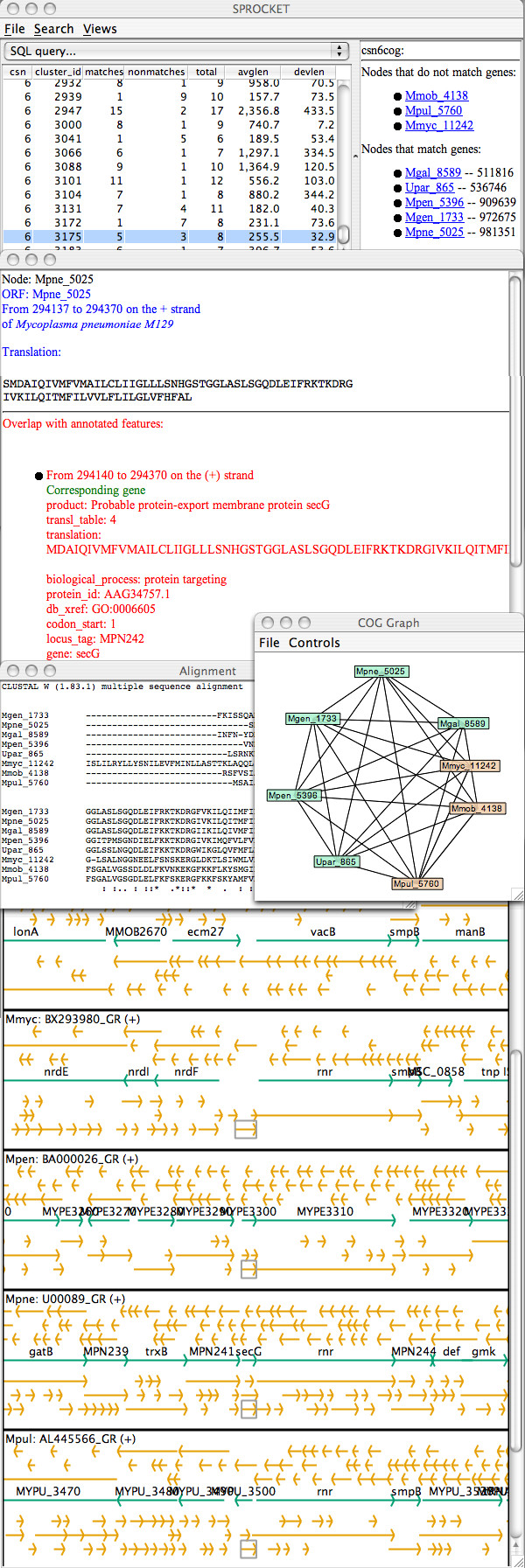
**SPROCKET**. The SPROCKET program was developed to facilitate the analysis performed in this study. For the members of a COG, a user can view the peptide sequence alignment (using CLUSTALW), a graph of the best-hit relationships and the genomic context.

The SPROCKET program (**S**ystem for **P**rotein **R**ecognition using **O**RF **C**OGs – a **K**nowledge **E**xtraction **T**ool) provides several ways of viewing and analyzing COG data of the type produced in this study (Figure 4). The front-end of the program is written in Java and runs on any platform with a version 1.4 or later Java virtual machine. Data for the program is stored in a relational database using a schema based on the BioSQL schema developed by the Open Bioinformatics Foundation [36] with extensions for the COG-specific information. Loading of initial sequences into the database was performed using portions of the BioJava project [37].

A summary view of the COGs shows the number of members of a COG and how many of those members share a stop codon with an annotated gene. Individual COGs can be examined in more detail by seeing a list of their members, a force-directed graph layout showing best-hit relationships among these members, an alignment of the potential peptide sequences of the ORFs as produced by CLUSTALW [38] or a graphical view of the regions of the genomes around the ORFs which are in a COG.

We provide source code for producing high stringency COGs [see Additional file 2] and for browsing the database of COGs [see Additional file 3].

## Availability and Requirements

Project name: SPROCKET

Project home page: 

Operating system(s): Platform independent

Programming language: C++ (COG construction), Java (COG viewing)

Other requirements: Java 1.4 or higher, PostgreSQL (only required if data stored locally)

License: GNU GPL (COG construction), BSD (COG viewing)

## Authors' contributions

BP conceived the study, wrote the software, analyzed the results and drafted the manuscript. CH supervised the study and provided extensive suggestions on the research as it progressed and on the manuscript. Both authors read and approved the final manuscript.

## Supplementary Material

Additional File 1 This Excel file contains the tabulated sensitivities, specificities, and positive predictive values for classification of open reading frames as genes or not genes, compared to existing annotations. Click here for file

Additional File 2 Source code archive for a C++ program to find high-stringency COGs given a set of bidirectional best-hit pairs. In gzip-compressed tar format. Click here for file

Additional File 3 Source code archive for a Java program to browse the database of COGs discussed in this manuscript. In gzip-compressed tar format. Click here for file

## Figures and Tables

**Table 1:** Genomes included in this study

Accession^a^	Name	Length (nt)	# of genes annotated^a^	# of ORFs^b ^>30 aa
BA000004	*Bacillus halodurans *strain C-125	4202352	4066	73839
BSXX	*Bacillus subtilis subsp. subtilis *str. 168	4214630	4106	75310
AE000783	*Borrelia burgdorferi *str. B31	910724	850	10756
AE001273	*Chlamydia trachomatis *strain D/UW-3/CX	1042519	894	17211
AE001363	*Chlamydophila pneumoniae *CWL029	1230230	1052	19259
AE001437	*Clostridium acetobutylicum *strain ATCC 824	3940880	3672	48244
BA000016	*Clostridium perfringens *str. 13	3031430	2660	31417
U00096	*Escherichia coli *K12	4639221	4289	86919
AE005174	*Escherichia coli *O157:H7 EDL933	5528970	5349	102747
L42023	Haemophilus influenzae Rd KW20	1830138	1709	27756
AE001439	*Helicobacter pylori *J99	1643831	1491	21997
AL591824	*Listeria monocytogenes *	2944528	2855	45146
AE015450	*Mycoplasma gallisepticum *str. R	996422	726	13506
L43967	*Mycoplasma genitalium *strain G-37	580074	480	8058
AE017308	*Mycoplasma mobile *strain 163K	777079	633	10241
BX293980	*Mycoplasma mycoides subsp. mycoides *SC	1211703	1016	14127
BA000026	*Mycoplasma penetrans *strain HF-2	1358633	1037	17111
U00089	*Mycoplasma pneumoniae *strain M129	816394	688	13868
MPUABCTIP	*Mycoplasma pulmonis *(Sabin 1941) Freundt 1955	963879	782	13324
AE002098	*Neisseria meningitidis *serogroup B strain MC58	2272351	2025	42660
AE004091	*Pseudomonas aeruginosa *str. PAO1	6264403	5566	92461
RPXX	*Rickettsia prowazekii *da Rocha-Lima 1916	1111523	834	12029
STYPHCT18	*Salmonella enterica subsp. enterica *serovar Typhi	4809037	4600	90974
AE007317	*Streptococcus pneumoniae *str. R6	2038615	2043	31733
AE000520	*Treponema pallidum subsp. pallidum *str. Nichols	1138011	1031	21937
AF222894	*Ureaplasma urealyticum *biovar 2	751719	611	9173
AE003852	*Vibrio cholerae *serotype O1 biotype ElTor strain N16961	2961149	2736	53378
AE003853	*Vibrio cholerae *serotype O1 biotype ElTor strain N16961	1072315	1092	19506

^a^Accessions and annotated genes reference Genome Reviews version 25.0

**Table 2:** ORFs in Majority-annotated mixed COGs of stringency 6 that may represent missed genes

ORF COG id^a^	Organism	Genomic coordinates^b^	Annotated gene(s) present in COG^c^	ORF COG id^a^	Organism	Genomic coordinates^b^	Annotated gene(s) present in COG^c^
	
Potential genes missed in current annotations				Potential genes missed in current annotations (continued)			
	
678	Bbur	117772-116825	*cdsA *	397	Nmen	340008-339358	*coaE *
314	Bhal	1503738-1503905	*rpmG *	871	Nmen	554238-552676	*mucD/deg *
314	Bsub	2477091-2476963	*rpmG *	723	Nmen	666433-665363	*potA/cysA/malK *
2346	Bsub	4202360-4202148		119	Nmen	690163-687386	*trkH *
1717	Cace	243535-242696	*alx *	1382	Nmen	1056138-1057340	*hflX *
1908	Cace	1395172-1395522	*minE *	464	Nmen	1147918-1149261	*tilS *
2064	Cace	2284461-2283778		464	Nmen	1179954-1181297	*tilS *
148	Cace	3287735-3286509	*tufA *	2743	Nmen	1400226-1401977	
1840	Cace	3650828-3649308		978	Nmen	1484110-1486353	*dnaX *
659	Cace	3842459-3840768	*plpB *	635	Nmen	1527781-1528521	
1551	EcoK12	311756-311598	*rpmJ *	1248	Nmen	1629570-1628017	*pepA *
148	EcoK12	3469408-3468167	*tufA *	2793	Nmen	1749455-1752016	*gcvP *
1551	EcoO157	344941-344783	*rpmJ *	618	Nmen	2119341-2120882	*hrpB *
2748	EcoO157	4240898-4240665		618	Nmen	2124720-2128169	*hrpB *
2531	Hinf	131970-132959	*mltA *	788	Nmen	2199859-2200686	*folD *
2319	Hinf	170676-169396	*dcuB *	2519	Paer	224101-225219	*ald *
2432	Hinf	235913-238519		1385	Paer	434829-433933	
2947	Hinf	370735-372912		38	Paer	4143744-4142569	*prfA *
1098	Hpyl	315887-316504	*dppC *	2748	Sent	4247574-4247864	
309	Lmon	640139-639558	*bioY *	192	Tpal	213049-213270	*rpmD *
2023	Mgen	180733-181020		653	Tpal	624206-625738	*ptsP *
994	Mmob	102995-102588	*nusB *	890	Tpal	946250-944889	*comM *
3131	Mmob	201807-201646	*rpmG *	946	Tpal	1032059-1031772	
3175	Mmob	317659-317411	*secG *	39	Upar	3002-3886	*hemK *
3186	Mmob	449811-451241		142	Upar	3861-4427	
3000	Mmyc	441031-441783		3131	Upar	725869-726024	*rpmG *
542	Mmyc	441031-441783		38	VchoI	709524-710558	*prfA *
199	Mmyc	830915-830742	*rpmI *	2932	VchoI	1045279-1044317	
73	Mmyc	831148-830924	*infA *	2947	VchoI	1627856-1625871	
182	Mmyc	836915-836712	*rpsN *	1246	VchoI	2869620-2871836	*pulA/glgX *
3175	Mmyc	973088-973423	*secG *	2793	VchoII	295059-292882	*gcvP *
3131	Mmyc	1089962-1090141	*rpmG *	2621	VchoII	299032-300000	*gcvT *
314	Mmyc	1089962-1090141	*rpmG *	2699	VchoII	406033-405167	*sbp *
1670	Mpen	2755-3009		2573	VchoII	987698-986424	*aroF/aroG/aroH *
3131	Mpen	1191375-1191163	*rpmG *	2340	VchoII	1026697-1023563	*dhaS/aldA *
879	Mpen	1226934-1226722	*rpmI *				
199	Mpen	1317088-1316960	*rpmI *	Gene annotated in different frame^d^			
166	Mpen	1327926-1326898	*rplV *	1769	Bhal	251734-251429	*nrdG *
2023	Mpne	207436-207717		3183	Mpul	130854-130480	
2090	Nmen	70930-70358		3175	Mpul	412829-413074	*secG *
148	Nmen	149590-150777	*tufA *	946	Rpro	433751-433479	
2564	Nmen	238562-237666		363	Tpal	262583-262897	*rpsT *
2572	Nmen	299359-298070	*phr *				

^a^The identifiers for COGs are local to this study. They do not correspond to numbers in the NCBI COG database.

^b^Coordinates in which the first number is greater than the second indicate that the ORF is on the minus strand.

^c^A named annotated putative ortholog in another organism or paralog within the organism to the ORF listed.

^d^These COGs may indicate both that the ORF listed is a missed gene and that the annotated

**Table 3:** ORFs in majority-annotated mixed COGs that do not appear to represent missed genes

ORF COG id^a^	Organism	Genomic coordinates^b^	Annotated gene(s) present in COG^c^	ORF COG id^a^	Organism	Genomic coordinates^b^	Annotated gene(s) present in COG^c^
	
Existing annotation of pseudogene				Frameshift 3' fragment^c^			
	
876	EcoK12	1488620-1487985	*gap *	1036	Bbur	21098-20445	*queA *
2340	Sent	4738725-4740071	*dhaS/aldA *	1750	Bhal	984866-983856	*celB *
2433	Sent	4745051-4743573	*hsdB *	1188	Bhal	1359362-1360555	*recD *
1895	Sent	3243737-3244861	*fadH *	2257	Bhal	3182850-3181696	*ilvI/poxB/alsS *
1399	Sent	461578-461874		88	Bsub	3671944-3672555	*gtaB *
653	Sent	2505700-2506824	*ptsP *	641	Hinf	1525427-1524561	*thiI *
1058	Sent	3413535-3416306	*acrD/mdtC/mdtB *	2031	Hinf	1719924-1718821	*tldD *
3088	Sent	4084807-4083605		2473	Mgal	431452-431778	*fldA *
815	Sent	1360931-1362226	*rhlE *	2309	Mgen	416785-416336	*acpD *
3104	Sent	4009730-4009993		975	Mmyc	57011-56760	*recR *
569	Sent	1969437-1970648	*penA *	686	Mmyc	690895-690356	*rpsB *
				1319	Nmen	107757-109406	*msbA *
Annotated in GenomeReviews but with different stop				842	Nmen	1995043-1994876	
928	Bsub	2500726-2499347	*bfmBC *	556	VchoI	553588-552383	*dnaG *
157	Cper	2751593-2751051	*rplD *	745	VchoI	555313-556182	*gcp *
999	EcoO157	3613249-3610595	*alaS *	106	VchoI	1087924-1089819	*uvrB *
107	Hinf	655042-654365	*metI *	2435	VchoI	2612949-2611972	
589	Mpne	329463-331229	*lepA *	2807	VchoII	1060889-1060107	*qseC *
210	Mpul	150772-151668	*grpE *				
743	Sent	2492196-2490763	*gltX *	Frameshift 5' fragment^c^			
534	Tpal	478406-478777		697	Bhal	3580443-3579682	*csd *
166	Upar	279005-279949	*rplV *	2029	Bsub	2304436-2305248	*metA *
				2049	Bsub	3032201-3032512	
Fragments around stop codons (nonsense)^c^				2	Cpne	383405-384037	*recF *
928	Bsub	2500726-2499347	*bfmBC *	462	Cpne	1088259-1088711	*ispE *
157	Cper	2751593-2751051	*rplD *	2769	EcoK12	3814680-3813886	*rph *
999	EcoO157	3613249-3610595	*alaS *	2257	EcoK12	3948538-3949566	*ilvI/poxB/alsS *
107	Hinf	655042-654365	*metI *	2433	Hinf	232074-232991	
589	Mpne	329463-331229	*lepA *	3066	Hinf	1377365-1378063	*dgt *
210	Mpul	150772-151668	*grpE *	1075	Hinf	1477189-1476557	*pstB *
743	Sent	2492196-2490763		641	Hinf	1526028-1525285	*thiI *
				2571	Nmen	292645-294051	
				220	Tpal	220772-221749	*dnaJ *
				556	VchoI	554244-553561	*dnaG *
				1826	VchoI	637551-638246	*amt *
				42	VchoI	851189-849954	*oadA *
				1082	VchoII	690599-690273	*glpF *

^a^The identifers for COGs are local to this study. They do not correspond to numbers in the NCBI COG database.

^b^Coordinates in which the first number is greater than the second indicate that the ORF is on the minus strand.

^c^A named annotated putative ortholog in another organism or paralog within the organism to the ORF listed.

^d^These categories represent probable pseudogenes or sequencing errors.

**Table 4:** Minority-annotated mixed COGs of stringency 6

^a^ORF COG id	Organism	^b^Genomic coordinates	Annotated locus tag	Explanation for similarity
458	Bhal	2607307-2607975	*BH2488 *	ambiguous--*smc *may be annotated as too long
2939	Lmon	2784312-2784674	*MYPU_4520 *	opposite strand *dnaG *
3041	Mgen	400107-399841	*MG320.1 *	opposite strand tRNA cluster
715	Mmob	474080-474634	*MMOB3820 *	opposite strand tRNA cluster
1171	Mmyc	315687-315178	*MSC_0275 *	opposite strand annotated gene
1172	Mmyc	315687-315178	*MSC_0275 *	opposite strand annotated gene
3172	Mpul	547792-547565	*LMO2711 *	opposite strand RNA-gene (*scr *) in Bhal, Bsub
169	Mpul	703396-704043	*MYPU_5820 *	ribosomal protein in opposite strand
1148	Mpul	706478-707455	*MYPU_5880 *	opposite strand ribosomal protein
1436	Spne	199207-198743	*SPR0193 *	opposite strand ribosomal protein
400	Tpal	321084-317926	*TP0304 *	region upstream of gene is opposite *pyrG *
625	Tpal	580802-581407	*TP0539 *	opposite strand *pgk *

^a^COG identifiers are local to this study.

^b^Coordinates in which the first number is greater than the second indicate that the ORF is on the minus strand.

**Table 5:** Mixed COGs containing ORFs from *Mycoplasma genitalium *that do not correspond to annotated genes

^a^**Genome coordinates**	**Strand**	^b^**COG id**	**Notes**
180733-181020	+	4-3347	Homologous to genes in 12 other organisms, some annotated as N-utilization substance
237114-237299	-	4-1487	Deletion of 'C' at 237175 joins this to the gene (MG199) annotated at 236591-237084. Together the joined fragments are similar to ribonuclease genes. [GenBank:AAGX01000004.1]
416336-416785	-	4-3943	Deletion of 'G' at 416710 joins this to fragment at 416661-416939. Together the joined fragments are similar to acyl carrier protein diesterases. [GenBank:AAGX01000016.1]
290638-291003	+	4-8314	Insertion of 'T' at 290983 joins this fragment to the gene (MG243) annotated at 290922-291326. Together the joined fragments are similar to hypothetical genes in *M. pneumonia *, *M. gallisepticum *, and *U. parvum *. [GenBank:AAGX01000005.1]

^a^Coordinates and insertions/deletions refer to [GenBank:NC_000908.1]

^b^COG identifiers are local to this study
