# Supplementary figures and images for: Similarity-based gene detection: using COGs to find evolutionarily-conserved ORFs
Source: BMC Bioinformatics. 2006 Jan 19;7:31. doi: 10.1186/1471-2105-7-31 (PMC1386717; doi:10.1186/1471-2105-7-31)

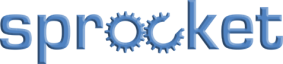

Supplement: Additional File 3 — Source code archive for a Java program to browse the database of COGs discussed in this manuscript. In gzip-compressed tar format. [file 1471-2105-7-31-S3.tgz › Sprocket-1.0/resources/edu/unc/med/diploid/Sprocket/utils/sprocket-logo-small.png]
